# Iatrogenic Hypoparathyroidism Development After Thyroidectomy: A Retrospective Cohort Study

**DOI:** 10.1002/edm2.506

**Published:** 2024-06-26

**Authors:** Amal A. Alomari, Raneen N. Abu Shanab, Randa A. Bajunaid, Lugean K. Alomari, Nidaa M. Almehmadi, Raghad S. Alzahrani, Alaa Althubaiti, Suhaib Radi

**Affiliations:** ^1^ College of Medicine King Saud Bin Abdulaziz University for Health Sciences Jeddah Saudi Arabia; ^2^ King Abdullah International Medical Research Center Jeddah Saudi Arabia; ^3^ Department of Endocrinology Ministry of National Guard Health – Affairs Jeddah Saudi Arabia

**Keywords:** hypocalcaemia, hypoparathyroidism, thyroidectomy

## Abstract

**Background:**

Iatrogenic hypoparathyroidism is a common cause of postthyroidectomy hypocalcaemia. It has varying incidence rates after neck surgery in Saudi Arabia, ranging from 0.07% to 65.30%. Hypoparathyroidism can manifest with a spectrum of symptoms, ranging from mild to severe and life‐threatening. This study aimed to assess the rate and predictors of iatrogenic hypoparathyroidism after thyroid surgery and its natural course.

**Methods:**

This retrospective cohort study used a data collection form to extract patient information from the electronic healthcare system (Best‐Care) for patients treated from 2017 to 2022. Patients' demographics, surgical specifics and biochemical profiles were recorded for subsequent analysis.

**Results:**

Among the 343 patients who underwent thyroidectomy, 130 (37.9%) developed hypoparathyroidism, primarily within the first day after surgery. Calcium or vitamin D supplementation before surgery did not significantly influence hypoparathyroidism development. Notably, extensive combined lymph node dissection was significantly associated with postoperative hypoparathyroidism development (*p* = 0.0004). More patients who underwent central and lateral lymph node dissection (*n* = 19, 79.17%) developed hypoparathyroidism than patients who underwent central (*n* = 18, 40.91%) or lateral (*n* = 8, 38.10%) dissection alone. Permanent hypoparathyroidism was observed in 40 patients (11.66%).

**Conclusion:**

This study revealed a high incidence of iatrogenic hypoparathyroidism and high rates of permanent hypoparathyroidism. Further research is warranted to better comprehend the risk factors and optimise management strategies for iatrogenic hypoparathyroidism. Overall, our findings emphasise the need for vigilant monitoring and effective management of patients undergoing thyroidectomy and the significance of postoperative replacement therapies.

## Graphical abstract

This retrospective cohort study aimed to assess the rate and predictors of iatrogenic hypoparathyroidism, a common complication of thyroid surgery. Among the 343 patients who underwent thyroidectomy, 37.9% developed hypoparathyroidism, with a significant association found between extensive combined lymph node dissection and its development. Permanent hypoparathyroidism was observed in 11.66% of the patients, highlighting the need for further research to understand the risk factors and improve management strategies for this condition. The study underscores the importance of careful monitoring and effective treatment for patients undergoing thyroid surgery.

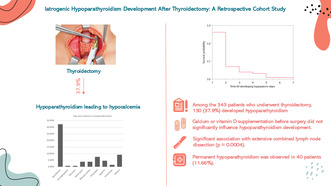

## 1 Introduction

The parathyroid gland, located in the posterior region of the thyroid gland, plays a vital role in the homeostasis of calcium in the bloodstream. Through the continuous secretion of parathyroid hormone (PTH) into the circulatory system, the parathyroid gland effectively regulates and maintains calcium levels within a narrow and precise range [1]. PTH exerts its primary influence by stimulating bone resorption, leading to the release of calcium into the bloodstream. Additionally, it reduces the excretion of calcium in the urine and exerts a modest impact on vitamin D metabolism, which contributes to the preservation of calcium levels [1, 2].

Hypoparathyroidism is characterised by abnormally low or absent PTH levels and hypocalcaemia and results from decreased parathyroid gland activity [1, 2, 3]. In addition, individuals with hypoparathyroidism often exhibit reduced 1,25‐dihydroxy vitamin D levels and elevated phosphorus levels (hyperphosphatemia) [2]. The aetiology of hypoparathyroidism can be broadly categorised into primary and secondary forms [3]. Primary hypoparathyroidism is attributed to autoimmune dysfunction or genetic abnormalities that occur during early development. In contrast, 75.00%–80.00% of hypoparathyroidism cases are classified as secondary or acquired, resulting from external factors that impair the parathyroid gland's function, including iatrogenic causes [2, 4, 5].

The primary and most common manifestation of hypoparathyroidism is hypocalcaemia, which occurs when there is insufficient calcium in the human body. This calcium deficiency affects various organ systems, leading to diverse symptoms in patients with hypoparathyroidism. These symptoms can vary in severity from mild manifestations such as tingling sensations (paraesthesia) and dry skin to severe and potentially life‐threatening symptoms such as seizures, laryngospasms (vocal cord spasms) and cardiac arrhythmias [6, 7].

In addition to the physical symptoms, hypoparathyroidism has also been associated with psychological disorders such as depression and anxiety [8]. Hypoparathyroidism can have chronic consequences over the long term, including the development of posterior subcapsular cataracts and calcification in the basal ganglia of the brain. Furthermore, patients undergoing calcium replacement therapy, which is often used to manage hypoparathyroidism, may experience complications such as excessive urinary calcium excretion (hypercalciuria) and the formation of calcium deposits in the kidneys (nephrocalcinosis) [9].

The incidence of transient hypoparathyroidism after neck surgery ranges from 25.80% to 83.00% worldwide, whereas permanent hypoparathyroidism occurs in about 0.12%–4.60% of cases [9]. The prevalence of hypoparathyroidism is approximately 37 cases per 100,000 individuals in the United States [9]. In Saudi Arabia, a retrospective study published in 2020 found that 67.40% of patients developed hypocalcaemia on the second day after total thyroidectomy [10]. Another study conducted in 2021 involving 1353 patients in seven Saudi provinces reported a prevalence of hypocalcaemia ranging from 0.07% to 65.30% [11].

Improving intraoperative identification and preservation of the parathyroid gland is crucial to minimise complications. Methylene blue is commonly used to locate the parathyroid gland during thyroid surgery, but its lack of specificity limits its widespread use [12]. Instead, 5‐aminolevulinic acid (5‐ALA) has become the preferred method. One study found that using 5‐ALA in patients who underwent thyroidectomy with lymphatic dissection reduced postsurgical hypoparathyroidism risk by 91.00% [13]. By enhancing parathyroid gland visualisation and mobilisation, 5‐ALA offers a more precise and effective approach to prevent complications during thyroid surgery.

As iatrogenic causes are prevalent among the various factors leading to hypoparathyroidism, this study's primary aim was to determine the rate of hypoparathyroidism after thyroid surgery. In addition, it aimed to identify the clinical, surgical and biochemical indicators that predict the development of iatrogenic hypoparathyroidism. Moreover, it aimed to evaluate the natural progression of hypoparathyroidism and assess its probability of becoming permanent.

## 2 Materials and Methods

### 2.1 Study Cohort

This retrospective cohort study involved 343 consecutive patients who underwent thyroidectomy for benign or malignant conditions at the main hospital of King Abdulaziz Medical City in Jeddah, Saudi Arabia, between 1 January 2017 and 31 December 2021. The study specifically targeted patients who were diagnosed with hypoparathyroidism. Hypoparathyroidism was defined as having a calcium level lower than 2.2 mmol/L, along with a low or inappropriately normal parathyroid hormone (PTH) level (10–15 pg/mL) on the first day following surgery. Patients with prior neck surgery or radiation, with other parathyroid diseases, or who underwent completion thyroidectomy were excluded from this study.

### 2.2 Data Collection

Participants' characteristics, including age, body mass index (BMI), comorbidities and smoking status, were collected from the electronic (Best‐Care) system using a data collection form for patients treated between 2017 and 2021. The surgery types performed, such as total thyroidectomy and hemithyroidectomy, were recorded, along with the reason for surgery, the number of identified and dissected lymph nodes and the number of identified and removed parathyroid glands. Preoperative and postoperative biochemical profiles, including vitamin D, calcium, phosphate and PTH levels, were also included in the analysis. Data on pre/postoperative supplements, such as vitamin D and calcium, were recorded. Outcome variables were recorded, such as the development of hypoparathyroidism symptoms, resolution of hypoparathyroidism, and duration of hospitalisation.

### 2.3 Statistical Analysis

Numerical data were described using parametric or nonparametric methods. Categorical data are presented as numbers (percentages). Outcomes were compared between patient groups using the chi‐squared or Fisher's exact test. Variables were compared between patient groups using the unpaired two‐tailed *t*‐test or two‐tailed Mann–Whitney *U* test. Overall survival was visualised using a Kaplan–Meier curve. Multiple logistic regression analysis was used to identify variables significantly and independently associated with hypoparathyroidism occurrence. Odds ratio (OR) with 95% confidence interval are reported. A *p* value of <0.05 was considered statistically significant. All statistical analyses were performed using JMP software (version 8.1; SAS Institute Inc., Cary, NC, USA).

## 3 Results

### 3.1 Participants' Characteristics

Table 1 presents the characteristics of the participants following thyroidectomy. Among the cohort of 343 participants, a total of 130 individuals (37.90%) experienced the development of hypoparathyroidism following the procedure. The majority of patients were female, with a count of 275 (80.17%), among whom 107 (38.91%) developed hypoparathyroidism. The study included 68 male participants (19.83%), and among them, 23 individuals (33.82%) developed hypoparathyroidism. Among the participants who developed hypoparathyroidism, 59 individuals (35.76%) had comorbidities such as diabetes mellitus (*n* = 26, 42.62%), hyperthyroidism (*n* = 5, 35.72%), hypertension (*n* = 28, 38.89%), ischemic heart disease (*n* = 2, 25.00%) or dyslipidaemia (*n* = 15, 41.67%). The average age of participants in the hypoparathyroidism group was 46.21 ± 16.11 years, whereas it was 44.81 ± 14.11 years in the nonhypoparathyroidism group (*p* = 0.59).

**TABLE 1:** Participants' characteristics.

Characteristic	Developing hypoparathyroidism		
	No (*n* = 213)	Yes (*n* = 130)	*p*
Age at diagnosis, mean ± SD, years	44.81 ± 14.11	46.21 ± 16.11	0.59
BMI, mean ± SD, kg/m^2^	29.31 ± 6.65	30.73 ± 6.81	0.06
Sex			
Male	45 (66.18)	23 (33.82)	0.44
Female	168 (61.09)	107 (38.91)	
Smoker			
Yes	16 (76.19)	5 (23.81)	0.17
No	197 (61.18)	125 (38.82)	
Comorbidity	106 (64.24)	59 (35.76)	0.43
DM	35 (57.38)	26 (42.62)	0.40
Hyperthyroidism	9 (64.29)	5 (35.72)	0.86
HTN	44 (61.11)	28 (38.89)	0.85
IHD	6 (75.00)	2 (25.00)	0.45
DLP	21 (58.33)	15 (41.67)	0.62
Autoimmune diseases	2 (66.67)	1 (33.33)	0.87
Cancer	8 (47.06)	9 (52.94)	0.19

*Note*: Data are presented as *n* (%) unless otherwise stated.

Abbreviations: DM, diabetes mellitus; DLP, dyslipoproteinaemia; HTN, hypertension; IHD, ischemic heart disease; SD, standard deviation.

Regarding the surgery type, out of the 130 patients who developed hypoparathyroidism, 123 underwent total thyroidectomy whereas only seven patients developed it after hemithyroidectomy (Table 2).

**TABLE 2:** Association between surgical factors, postoperative outcome and hypoparathyroidism development.

	Developing hypoparathyroidism		*p*
	No	Yes	
Number of parathyroid glands removed			
None	174 (62.59)	104 (37.41)	0.25
1	24 (68.57)	11 (31.43)	
≥2	12 (48.00)	13 (52.00)	
Number of parathyroid glands intraoperatively identified			
None	41 (70.69)	17 (29.31)	0.21
1	35 (66.04)	18 (33.96)	
≥2	132 (58.93)	92 (41.07)	
Reason for surgery			
Thyroid cancer	63 (50.00)	63 (50.00)	0.004
Benign condition	150 (69.12)	67 (30.88)	
Surgery type			
Total thyroidectomy	125 (50.40)	123 (49.60)	<0.0001
Hemithyroidectomy	88 (92.63)	7 (7.37)	
Lymph node dissection			
None	154 (65.53)	81 (34.47)	0.0004
Central	26 (59.09)	18 (40.91)	
Lateral	13 (61.90)	8 (38.10)	
Central and lateral	5 (20.83)	19 (79.17)	
Hospitalisation duration, median (interquartile range), days	3 (2)	6 (6)	<0.0001

*Note*: Data are presented as *n* (%) unless otherwise stated.

Abbreviation: MNG, multinodular goitre.

### 3.2 The Association of Pre/Postoperative Biochemical Profile With Iatrogenic Hypoparathyroidism

There was no significant correlation found between the preoperative levels of calcium (*p =* 0.15), phosphorus (*p =* 0.30), magnesium (*p =* 0.14), PTH (*p* = 0.46) and vitamin D (*p =* 0.46) and the occurrence of hypoparathyroidism. However, in the postoperative period, there was a significant association between these laboratory values and the development of hypoparathyroidism, with the exception of vitamin D deficiency (*p* = 0.59), which did not show a significant association with the development of hypoparathyroidism (Table 3).

**TABLE 3:** Association of pre‐ and postoperative laboratory tests and preoperative replacement therapy with hypoparathyroidism development.

	Developing hypoparathyroidism		
	No	Yes	*p*
Preoperative laboratories, median (interquartile range)			
Ca	2.29 (0.11)	2.27 (0.11)	0.15
PO_4_	1.14 (0.25)	1.26 (0.26)	0.30
Mg	0.79 (0.09)	0.76 (0.12)	0.14
PTH	76.20 (48.55)	73.60 (69.70)	0.79
Vitamin D	36.30 (28.35)	38.45 (44.30)	0.46
TSH	1.53 (1.28)	1.54 (1.69)	0.70
Postoperative laboratories, median (interquartile range)			
Ca	2.25 (0.14)	2.14 (0.17)	<0.0001
PO_4_	1.19 (0.33)	1.29 (0.34)	0.0087
Mg	0.75 (0.15)	0.69 (0.12)	<0.0001
PTH	46.70 (39.70)	8.50 (30.50)	<0.0001
Vitamin D	38.40 (28.80)	43.05 (40.88)	0.59
Preoperative replacement therapy, *n* (%)			
Ca replacement	38 (58.33)	25 (41.67)	0.06
Vitamin D replacement	32 (51.61)	30 (48.39)	0.06
Active vitamin D replacement	23 (58.97)	16 (41.03)	0.67
Postoperative replacement therapy, *n* (%)			
Ca replacement	133 (51.15)	127 (48.86)	<0.0001
Vitamin D replacement	44 (44.90)	54 (55.10)	<0.0001
Active vitamin D replacement	140 (55.12)	114 (44.88)	<0.0001

Abbreviations: Ca, calcium; Mg, magnesium; PO_4_, phosphate; PTH, parathyroid hormone; TSH, thyroid stimulating hormone.

### 3.3 The Association of Pre/Postoperative Replacement Therapy With Iatrogenic Hypoparathyroidism

In terms of preoperative replacement therapies, there was no significant difference in the incidence of hypoparathyroidism between participants who received calcium, vitamin D and active vitamin D supplementation before surgery and those who did not (Table 3).

Regarding postoperative vitamin D replacement, 55.1% of those who received inactive vitamin D developed hypoparathyroidism (*p* < 0.001), whereas only 44.88% of those who received active vitamin D replacement developed hypoparathyroidism (*p* < 0.001) (Table 3).

The majority of iatrogenic hypoparathyroidism patients did not need PTH replacement therapy using PTH analogues, whereas only one patient (1%) required it. Additionally, 48 participants (37%) required intravenous calcium replacement, as indicated in Table S1. The need for intravenous calcium replacement therapy was significantly associated with the type of surgery performed (*p* < 0.0001). Almost all participants who required intravenous calcium replacement underwent total thyroidectomy (*n =* 51 out of 52) whereas only one participant who underwent hemithyroidectomy required it, as shown in Table S2.

### 3.4 Surgical Approach and Developing Hypoparathyroidism

Although hypoparathyroidism tended to develop more often when more glands were removed (Table 2), the trend was nonsignificant (*p* = 0.25). However, extensive combined lymph node dissection was significantly associated with postoperative hypoparathyroidism development (*p* = 0.0004). Specifically, most participants who underwent central and lateral lymph node dissection developed hypoparathyroidism (*n* = 19, 79.17%; *p* = 0.0004), whereas fewer participants who underwent central lymph node dissection alone (*n* = 18, 40.91%) or lateral lymph node dissection alone (*n* = 8, 38.10%) developed hypoparathyroidism (Table 2).

### 3.5 Postoperative Outcomes

**FIGURE 1:**
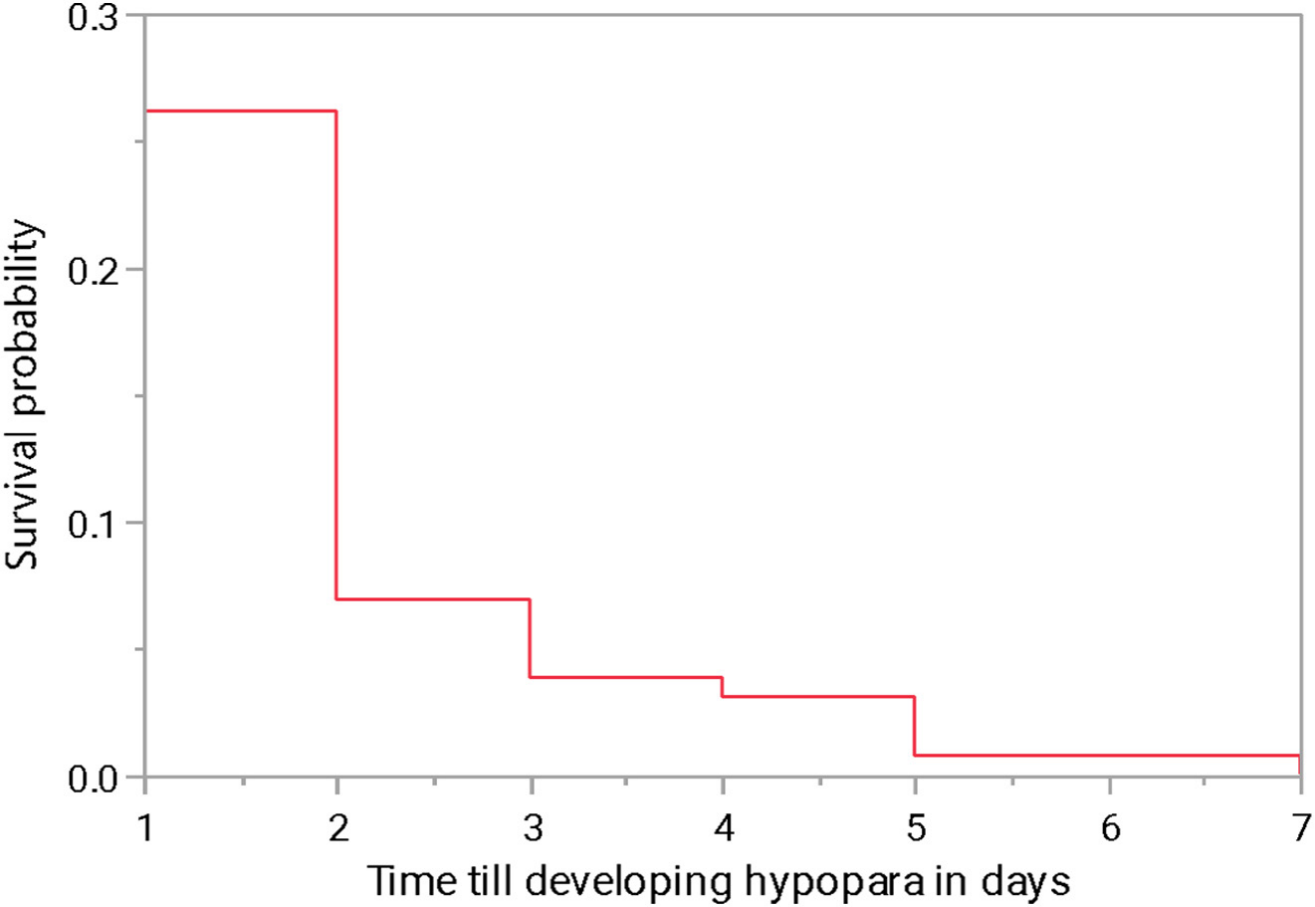
Time to hypoparathyroidism development in days.

**FIGURE 2:**
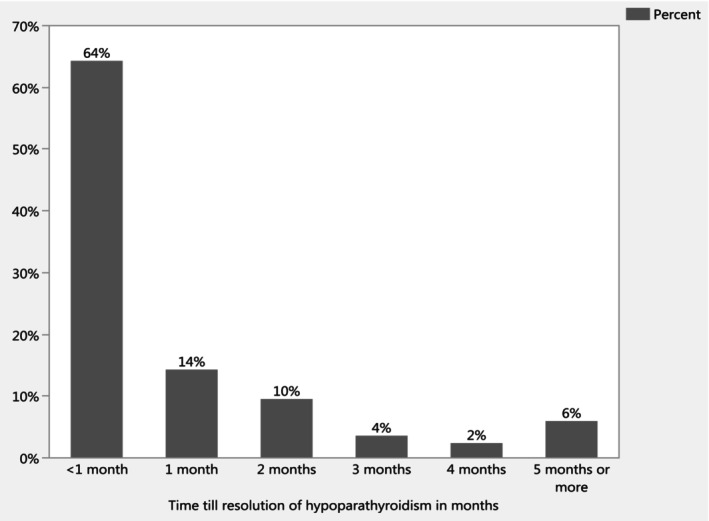
Time to hypoparathyroidism resolution in months.

Figure 1 shows that most participants who developed hypoparathyroidism were diagnosed 1 day after surgery. Among the 130 participants with hypoparathyroidism, 58 (45.67%) showed its signs and/or symptoms. The most common sign and symptom was numbness (*n* = 42, 32.31%), followed by chvostek sign (*n* = 10, 7.69%; Table S3). Additionally, hypoparathyroidism development was associated with a significantly longer hospitalisation period (*p* < 0.0001). Participants with iatrogenic hypoparathyroidism were hospitalised for a median of 6 days with an interquartile range of 6, whereas those who underwent noncomplicated thyroid surgeries had a median hospitalisation period of 3 days with an interquartile range of 2 (Table 2). Approximately two‐thirds of the participants with hypoparathyroidism (*n* = 90, 69.23%) achieved resolution, primarily within 1 month (Figure 2). In contrast, 40 participants out of the total cohort (11.66%) developed permanent hypoparathyroidism as shown in Table S1.

### 3.6 Hypoparathyroidism Resolution

Hypoparathyroidism resolution differed significantly between participants who underwent total thyroidectomy and those who underwent hemithyroidectomy (*p* = 0.013). Hypoparathyroidism resolved in most patients who underwent total thyroidectomy (*n* = 83, 67.48%) and in all patients who underwent hemithyroidectomy (*n* = 7, 100%). Moreover, hypoparathyroidism resolved in most participants who had none of their lymph nodes dissected (*n* = 63, 73.26%) or had none of their parathyroid glands removed (*n* = 77, 72.64%), however, this did not reach statistical significance. Furthermore, hypoparathyroidism resolution was more common in male (*n* = 18, 85.71%) than in female (*n* = 72, 66.05%) participants (*p* = 0.024) (Table S4).

The regression analysis indicated that surgery type was independently and significantly associated with hypoparathyroidism development. Participants who underwent total thyroidectomy were more likely to develop hypoparathyroidism than those who underwent hemithyroidectomy (OR = 12.3, 95% confidence interval = 5.5–27.8, *p* < 0.0001) (Table S5).

## 4 Discussion

Hypoparathyroidism is a known but dreaded complication of thyroid surgery due to the close anatomical proximity of the parathyroid glands to the thyroid gland. Our study, comprising 343 patients who underwent thyroid surgery, offers several valuable insights into the incidence and risk factors associated with postthyroidectomy hypoparathyroidism. The incidence of postsurgical hypoparathyroidism was 37.90%, which is on the higher end but within the range reported by other studies [5, 14]. Two‐thirds of the participants who developed hypoparathyroidism recovered, mainly within the first month. However, their rate of permanent hypoparathyroidism (beyond 6 months) was elevated (11.66%) compared with the literature (2%–4%) [14].

The markedly higher incidence of hypoparathyroidism among patients who underwent total thyroidectomy (49.60%) than hemithyroidectomy (7.37%) suggests that the extent of surgical intervention is a significant risk factor. This observation is further supported by the regression analysis, which demonstrated a statistically significant association between the surgery type and the likelihood of developing hypoparathyroidism. Furthermore, the correlation between extensive combined lymph node dissection and higher rates of hypoparathyroidism indicates that increased surgical manipulation or potential damage to the blood supply of the parathyroid glands can elevate the risk. The hypoparathyroidism diagnosis was significantly associated with combined lymph node dissection (*p* = 0.0004), consistent with similar findings reported by Ru et al. [15]. The diverse aetiologies that necessitate thyroid surgical intervention should be considered. In our study, multinodular goitre (MNG) was the most common reason for thyroidectomy, whereas malignancy was the most commonly associated indication for developing iatrogenic hypoparathyroidism. Conversely, Ritter et al. [5] found that thyroidectomy due to benign conditions was the primary cause of postoperative hypoparathyroidism, followed by cancer.

Preoperative vitamin D or calcium supplementation was not significantly associated with postoperative hypoparathyroidism development. However, patients receiving active vitamin D replacement showed a lower risk of developing hypoparathyroidism than those receiving inactive vitamin D supplements. This observation had intriguing therapeutic implications, although our study's design limits our ability to determine whether patients received postoperative replacement before or after hypoparathyroidism onset. A randomised controlled trial conducted in 2021 investigated the effects of cholecalciferol, an inactive form of vitamin D, on the rate of permanent hypoparathyroidism. It reported a lower incidence of permanent hypoparathyroidism in those receiving cholecalciferol, although the difference was nonsignificant [16]. Furthermore, a meta‐analysis by Sanabria et al. concluded that routine postoperative active vitamin D replacement effectively reduced hypocalcaemia development [17]. These findings further support the potential benefits of vitamin D supplementation in managing hypoparathyroidism after surgery.

An additional finding of our study was the association between hypoparathyroidism and hypomagnesemia in patients who underwent thyroidectomy. A 2018 study similarly reported that 34% of patients who underwent thyroidectomy developed hypomagnesemia [18]. Hypomagnesemia may contribute to low PTH levels, and correcting magnesium levels could help improve PTH levels in these patients.

Hypoparathyroidism was most frequently diagnosed 1 day after surgery, highlighting the importance of early and vigilant postoperative monitoring for potential complications. A significant observation from our study was the extended hospital stay of patients who developed iatrogenic hypoparathyroidism. The hospitalisation duration was 3 days longer for those diagnosed with hypoparathyroidism, underscoring its clinical impact and emphasising the importance of early detection and intervention.

Our study found several factors to favour hypoparathyroidism resolution. Patients who underwent hemithyroidectomy had a 100% resolution rate, indicating a higher likelihood of recovery than those treated with other surgical procedures. Additionally, patients who did not undergo lymph node or parathyroid gland dissection had a better resolution rate. Interestingly, male patients showed higher hypoparathyroidism resolution than female patients, suggesting the need for further investigation to uncover underlying factors or potential protective mechanisms.

Our study is subject to certain limitations that warrant consideration. Firstly, the retrospective collection of data from a single centre introduces the potential for institutional bias and may limit the generalizability of our findings to other healthcare settings and also some instances of missing data could have influenced the completeness and accuracy of our analysis. Therefore, it is important to exercise caution when interpreting our results and extrapolating them to clinical practice.

In conclusion, our study has shed light on the critical factors that influence the occurrence and resolution of hypoparathyroidism following thyroidectomy. Our findings highlight the significant impact of surgical procedure type, biochemical parameters and postoperative interventions. Of particular concern is the notable incidence of permanent hypoparathyroidism observed in our cohort. To gain a more comprehensive and robust understanding of this complication, further research conducted prospectively and involving multiple centres is warranted.

Furthermore, there is a pressing need to prioritise patient and clinician education regarding early symptom recognition and the importance of diligent postoperative monitoring. By enhancing awareness and implementing proactive measures, we can effectively mitigate the long‐term consequences associated with hypoparathyroidism.

## Author Contributions

**Amal A. Alomari:** data curation (equal), formal analysis (equal), investigation (equal), writing–original draft (equal), writing–review and editing (equal). **Raneen N. Abu Shanab:** data curation (equal), investigation (equal), writing–original draft (equal). **Randa A. Bajunaid:** data curation (equal), investigation (equal), writing–original draft (equal). **Lugean K. Alomari:** data curation (equal), investigation (equal), writing–original draft (equal). **Nidaa M. Almehmadi:** data curation (equal), investigation (equal), writing–original draft (equal). **Raghad S. Alzahrani:** data curation (equal), formal analysis (equal), writing–original draft (equal), writing–review and editing (equal). **Alaa Althubaiti:** data curation (equal), formal analysis (lead), methodology (equal), supervision (supporting), writing–review and editing (equal). **Suhaib Radi:** conceptualization (lead), methodology (equal), project administration (lead), supervision (lead), writing–review and editing (equal).

## Ethics Statement

Our ethics committee at King Abdullah International Medical Research Center in Jeddah granted approval for this study under the institutional review board (IRB) number: JED‐22‐427780‐53916. As it was a retrospective study, obtaining consent forms was not necessary.

## Conflicts of Interest

The authors declare no conflicts of interest.

## Supporting information


Table S1.
Table S2.
Table S3.
Table S4.
Table S5.
Table S6.

## Data Availability

The manuscript and supporting information contain all available data for this study.
