# Supplementary material for: Iatrogenic Hypoparathyroidism Development After Thyroidectomy: A Retrospective Cohort Study
Source: Endocrinol Diabetes Metab. 2024 Jun 26;7(4):e506. doi: 10.1002/edm2.506 (PMC11208280; doi:10.1002/edm2.506)
Supplement: Supplementary file 1 — Table S1. Table S2. Table S3. Table S4. Table S5. Table S6. [file EDM2-7-e506-s001.docx]

**SUPPLEMENTAL TABLE 1:**  Postoperative outcomes.

|  | Hypoparathyroidism group  (*n* = 130) |
| --- | --- |
| Need for teriparatide (PTH analogs) | 1 (1%) |
| Need for IV calcium replacement | 48 (37%) |
| Hypoparathyroidism resolution | 90 (69.23%) |
| Time to hypoparathyroidism development, median (interquartile range), days | 1 (1) |

Data are presented as *n* (%) unless otherwise stated.

**SUPPLEMENTAL TABLE 2:** Association of reason for surgery, number of parathyroid glands removed and identified, and surgery type with the need for intravenous calcium replacement.

|  | **Need for IV calcium replacement** | | |  |
| --- | --- | --- | --- | --- |
|  | **No**  **(*n* = 291)** | **Yes**  **(*n* = 52)** | | ***p*** |
| **Reason for surgery** |  |  |  |  |
| Thyroid cancer | 97 (76.98) | 29 (23.02) | | 0.0002 |
| Benign condition | 194 (89.40) | 23 (10.60) | |  |
| **Number of parathyroid glands removed** |  |  | |  |
| None | 239 (85.97) | 39 (14.03) | | 0.057 |
| 1 | 30 (85.71) | 5 (14.29) | |  |
| ≥2 | 17 (68) | 8 (32) | |  |
| **Number of parathyroid glands intraoperatively identified** |  |  | |  |
| None | 50 (86.21) | 8 (13.79) | | 0.94 |
| 1 | 45 (84.91) | 8 (15.09) | |  |
| ≥2 | 189 (84.38) | 35 (15.63) | |  |
| **Surgery type** |  |  | |  |
| Total thyroidectomy | 197 (79.44) | 51 (20.56) | | < 0.0001 |
| Hemithyroidectomy | 94 (98.95) | 1 (1.05) | |  |

Data are presented as *n* (%).

**SUPPLEMENTAL TABLE 3:** Signs and symptoms of hypoparathyroidism.

| Signs and symptoms | *n* (%) |
| --- | --- |
| Any sign or symptom | 58 (45.67) |
| Numbness | 42 (32.31) |
| Laryngospasm | 1 (0.77) |
| Arrhythmia | 0 (0) |
| Seizures | 1 (0.77) |
| Bone pain | 5 (3.85) |
| Muscle aches | 5 (3.85) |
| Chvostek | 10 (7.69) |
| Spasms | 6 (4.62) |
| Trousseau | 2 (1.54) |
| Other | 12 (9.23) |

**SUPPLEMENTAL TABLE 4:** The relationship between hypoparathyroidism resolution and other factors.

|  | **Hypoparathyroidism resolved** | |  |
| --- | --- | --- | --- |
|  | **No**  **(*n =* 40)** | **Yes**  **(*n* = 90)** | ***P*** |
| **Surgery type** |  |  |  |
| Total thyroidectomy | 40 (32.52) | 83 (67.48) | 0.013 |
| Hemithyroidectomy | 0 (0) | 7 (100) |  |
| **Lymph node dissection** |  |  |  |
| None | 23 (26.74) | 63 (73.26) | 0.71 |
| Central | 8 (44.44) | 10 (55.56) |  |
| Lateral | 2 (28.57) | 5 (71.43) |  |
| Central and lateral | 7 (36.84) | 12 (63.16) |  |
| **Sex** |  |  |  |
| Male | 3 (14.29) | 18 (85.71) | 0.024 |
| Female | 37 (33.95) | 72 (66.05) |  |
| **Number of parathyroid glands removed** |  |  |  |
| None | 29 (27.36) | 77 (72.64) | 0.28 |
| 1 | 4 (36.36) | 7 (63.64) |  |
| ≥2 | 7 (53.85) | 6 (46.15) |  |
| **Comorbidities** | 18 (31.58) | 39 (68.42) | 0.55 |
| **Preoperative Ca replacement** | 10 (40) | 15 (60) | 0.51 |
| **Preoperative vitamin D replacement** | 12 (40) | 18 (60) | 0.46 |
| **Preoperative active vitamin D replacement** | 5 (31.25) | 11 (68.75) | 0.77 |

Data are presented as *n* (%) unless otherwise stated.

**SUPPLEMENTAL TABLE 5:** Regression analysis.

| **Variables** | **OR** | **95%CI** | ***P*** |
| --- | --- | --- | --- |
| **Surgery type** |  |  |  |
| **Total thyroidectomy** | 12.3 | 5.5-27.8 | <0.0001 |

Reference group: hemithyroidectomy. OR. Odds ratio; CI, confidence interval.

**SUPPLEMENTAL TABLE 6:** Association between hypoparathyroidism resolution and laboratory values.

|  | **Hypoparathyroidism resolved** | |  |
| --- | --- | --- | --- |
|  | **No**  **(*n* = 40)** | **Yes**  **(*n* = 90)** | ***P*** |
| Preoperative labs (Ca) | 2.28 (0.11) | 2.27 (0.12) | 0.88 |
| Preoperative labs (PO_4_) | 1.12 (0.28) | 1.17 (0.27) | 0.37 |
| Preoperative labs (Mg) | 0.77 (0.16) | 0.75 (0.11) | 0.54 |
| Preoperative labs (PTH) | 69 (65.60) | 76 (67.43) | 0.91 |
| Preoperative labs (Vit D) | 33.6 (20.50) | 40.7 (50.30) | 0.20 |
| Preoperative labs (TSH) | 1.48 (1.43) | 1.54 (2.07) | 0.83 |

Data are presented as median (interquartile range). Ca: calcium; PO_4_: phosphate; Mg: magnesium; PTH: parathyroid hormone; Vit D: Vitamin D; TSH: thyroid stimulating hormone.
